# *DUXAP8*, a pseudogene derived lncRNA, promotes growth of pancreatic carcinoma cells by epigenetically silencing CDKN1A and KLF2

**DOI:** 10.1186/s40880-018-0333-9

**Published:** 2018-10-26

**Authors:** Yifan Lian, Jiebin Yang, Yikai Lian, Chuangxing Xiao, Xuezhen Hu, Hongzhi Xu

**Affiliations:** 1 Department of Gastroenterology, Zhongshan Hospital, Xiamen University, Xiamen, 361005 Fujian P. R. China; 2 Institute for Microbial Ecology, Xiamen University, Xiamen, 361005 Fujian P. R. China; 3 Jiangsu Province Hospital of TCM, Affiliated Hospital of Nanjing University of TCM, Nanjing, 210029 Jiangsu P. R. China

**Keywords:** Pancreatic cancer, Proliferation, Pseudogene, DUXAP8, CDKN1A, KLF2

## Abstract

**Background:**

Recent studies highlight pseudogene derived long non-coding RNAs (lncRNAs) as key regulators of cancer biology. However, few of them have been well characterized in pancreatic cancer. Here, we aimed to identify the association between pseudogene derived lncRNA *DUXAP8* and growth of pancreatic cancer cells.

**Methods:**

We screened for pseudogene derived lncRNAs associated with human pancreatic cancer by comparative analysis of three independent datasets from GEO. Quantitative real-time reverse transcription polymerase chain reaction was used to assess the relative expression of DUXAP8 in pancreatic cancer tissues and cells. Loss-of-function approaches were used to investigate the potential functional roles of DUXAP8 in pancreatic cancer cell proliferation and apoptosis in vitro and in vivo. RNA immunoprecipitation, chromosome immunoprecipitation assay and rescue experiments were performed to analyze the association of DUXAP8 with target proteins and genes in pancreatic cancer cells.

**Results:**

Pancreatic cancer tissues had significantly higher DUXAP8 levels than paired adjacent normal tissues. High DUXAP8 expression was associated with a larger tumor size, advanced pathological stage and shorter overall survival of pancreatic cancer patients. Moreover, silencing DUXAP8 expression by siRNA or shRNA inhibited pancreatic cancer cell proliferation and promoted apoptosis in vitro and in vivo. Mechanistic analyses indicated that DUXAP8 regulates PC cell proliferation partly through downregulation of tumor suppressor CDKN1A and KLF2 expression.

**Conclusion:**

Our results suggest that tumor expression of pseudogene derived lncRNA DUXAP8 plays an important role in pancreatic cancer progression. DUXAP8 may serve as a candidate biomarker and represent a novel therapeutic target of pancreatic cancer.

**Electronic supplementary material:**

The online version of this article (10.1186/s40880-018-0333-9) contains supplementary material, which is available to authorized users.

## Background

Pancreatic cancer currently remains one of the most lethal malignancies of the digestive system [1]. In developed countries, it was estimated that 187,500 people were diagnosed with pancreatic cancer in 2012, and 184,400 succumbed to the disease [2]. Progresses in several fronts including early detection, surgical intervention, systemic chemotherapy and targeted therapy have led to further improvements in the treatment of pancreatic cancer [3], but has not been translated into clinical benefits: the five-year overall survival (OS) rate of pancreatic cancer patients remains at only 5% [4]. The dismal outcome of pancreatic cancer patients highlights the importance of understanding the molecular mechanisms underlying progression of pancreatic cancer, which so far remain poorly defined. Therefore, it would be of great clinical relevance and significance to identify novel diagnostic and/or prognostic biomarkers associated with progression of pancreatic cancer.

Whole genome and transcriptome sequencing technologies have gradually shed light on the makeup of the human genome, less than 2% of which are protein coding genes with the remaining being non-coding genes including pseudogenes, microRNA genes, and long non-coding RNA (lncRNA) genes [5, 6]. Pseudogenes are the results of gene duplications, with the duplicated genes having lost their protein-coding capacity through such molecular events as point or frameshift mutations [6, 7]. Intriguingly, it is becoming increasingly apparent that many pseudogenes are transcribed into long non-coding RNAs, with confirmed biological functions in transcriptional or post-transcriptional regulation of gene expression by functioning as scaffold for distinct protein complexes or endogenous competitors for microRNA (miRNA) [8]. Several studies have demonstrated the biological roles of certain classical pseudogenes, including *POU5F1B*, *PTENP1* and *DUXAP10* in human cancers [9–11]. However, similar to lncRNAs, the functions of pseudogenes in pancreatic cancer require further investigation.

Previous studies indicated that pseudogenes derived lncRNAs could also regulate gene expression through other mechanisms, such as binding with certain RNA-binding proteins, as well as regulating target genes by lncRNAs [12, 13]. For example, Wei et al. [11] reported that pseudogene *DUXAP10* promotes cell proliferation and invasion through interacting with LSD1 and repressing LATS2 and RRAD in non-small-cell lung cancer. Guo et al. reported that pseudogene *PTENP1* suppresses gastric cancer aggressive phenotype by modulating PTEN expression [14].

In the current study, we sought to investigate the association between DUXAP8 expression and OS of pancreatic cancer patients and further delineate the effects of DUXAP8 on the growth of pancreatic cancer cells both in vitro and in vivo and the underlying mechanism. This study provides the first direct evidence that DUXAP8 is a critical and powerful regulator of genes involved in pancreatic cancer progression through silencing CDKN1A and KLF2 in the nucleus, indicating that DUXAP8 is a potential therapeutic target of pancreatic cancer.

## Methods

### Ethics statement

The study protocol was approved by the Research Ethics Committee of Xiamen University, Xiamen, Fujian, China. Written informed consent form was obtained from all patients. The handling of human tissue specimens was in full accordance with the relevant institutional and national guidelines and regulations. All patient data were de-identified for analysis and anonymized. Animal study was carried out in strict accordance with the USA Guide for the Care and Use of Laboratory Animals of the National Institutes of Health.

### Microarray and computational analysis

Three human microarray data sets including GSE16515, GSE15932 and GSE15471 were downloaded from the Gene Expression Omnibus (GEO) database (http://www.ncbi.nlm.nih.gov/geo) and normalized using Robust Multichip Average (RMA). The probe sequences were downloaded from GEO, and bowtie was used to re-annotate probes according to the GENCODE Release 19 annotation for pseudogenes or lncRNAs.

### Tissue acquisition

A total of 58 treatment-naïve patients undergoing resection of pathologically confirmed pancreatic ductal adenocarcinoma (PDAC) at Zhongshan Hospital, Xiamen University, Xiamen, Fujian, China between 2007 and 2012, were included in the study. All tissue samples were snap frozen in liquid nitrogen and stored at − 80 °C until use. The clinicopathologic characteristics of the patients are summarized in Table 1.

**Table 1:** Correlation between DUXAP8 expression and clinicopathologic characteristics of patients with PDAC

Characteristics	N	Percent (%)	DUXAP8 level		*P*
			Low	High	Chi squared test
Total cases	58	100	29	29	
Gender					0.790
Male	34	58.6	16	18	
Female	24	41.4	13	11	
Age (years)					1.000
< 65	29	50.0	14	15	
≥ 65	29	50.0	15	14	
Tumor size					0.033
< 3 cm	25	43.1	17	8	
≥ 3 cm	33	56.9	12	21	
Differentiation					0.289
Well/moderate	25	43.1	15	10	
Poor	33	56.9	14	19	
TNM stage					0.034
I/II	27	46.6	18	9	
III/IV	31	53.4	11	20	
Lymph-node metastasis					0.065
Negative	30	51.7	19	11	
Positive	28	48.3	10	18	

### Quantitative reverse transcription polymerase chain reaction (qRT-PCR) assays

Total cellular RNA was extracted from pancreatic cancer cells or tissues using TRIZOL reagent (Invitrogen, Carlsbad, CA, USA). RNA quantity was determined by NanoDrop2000c (Thermo Scientific, Waltham, MA, USA). For qRT-PCR, 1 μg RNA was reverse transcribed to cDNA using a Reverse Transcription Kit (Takara, Dalian, China). The qRT-PCR assays were conducted on an ABI 7500. Target gene expression was normalized against *GAPDH*. The primer sequences are listed in Additional file 1: Table S1.

### Cells

Pancreatic cancer cells (AsPC-1, BxPC-3, and PANC-1) and normal human pancreatic HPDE6 cell lines were obtained from the Institute of Biochemistry and Cell Biology of the Chinese Academy of Sciences (Shanghai, China), and maintained in RPMI 1640 or DMEM (Invitrogen, Shanghai, China) supplemented with 10% fetal bovine serum (FBS, HyClone, Camarillo, CA, USA), 100 U/mL penicillin, and 100 mg/mL streptomycin (Invitrogen) at 37 °C with 5% CO_2_.

### Transfections

Typically, pancreatic cancer cells were seeded at 5 × 10^5^ cells per well in six-well plates and then transfected on the next day with appropriate specific small interfering RNA (siRNA) (100 nmol/L) or scrambled control siRNA (100 nmol/L) using Lipofectamine 2000 (Invitrogen). Forty-eight hours after transfection, the cells were harvested for further experiments. The primer sequences and siRNA sequences are listed in Additional file 1: Table S1.

### Cell viability and colony formation assays

AsPC-1, BxPC-3 and PANC-1 cells were seeded at 3000 cells/well in 96-well plates and transfected with specific or control siRNA. Cell viability was assessed using the Cell Proliferation Reagent Kit I (MTT; Roche Applied Science). Each experiment was conducted at least three times independently in quintuplicate.

For colony formation assays, a total of 500 cells were seeded in a six-well plate and maintained in medium containing 10% FBS. The medium was replenished every 3 days. After 2 weeks, cells were fixed with methanol and stained with 0.1% crystal violet (Sigma Aldrich, St. Louis, MO, USA). Visible colony formation was determined by counting the number of stained colonies. An aggregate consisting of 50 cells or more was considered a colony and scored. Each experiment was conducted at least three times independently in triplicate.

### Flow cytometry

BxPC-3 and PANC-1 cells were transfected with appropriate specific or control siRNA and subsequently stained with propidium iodide using the Cycle TESTTM PLUS DNA Reagent Kit (BD Biosciences). The DNA content of the cells was analyzed by flow cytometry (FACScan^®^; BD Biosciences) equipped with the Cell Quest software (BD Biosciences). The percentage of cells in the G0-G1, S, or G2-M phase was calculated based on relative DNA content.

For analysis of apoptosis, BxPC-3 and PANC-1 cells were harvested 48 h post transfection. The cells were stained with fluorescein isothiocyanate (FITC) annexin V and propidium iodide in the dark at room temperature. Then, the cells were analyzed by FACScan^®^ and counted as viable, early apoptotic, late apoptotic, or dead cells.

### Xenograft assays

Four-week-old male athymic mice were purchased from the Animal Center of Xiamen University (Xiamen, China) and maintained in laminar flow cabinets under specific pathogen-free conditions. BxPC-3 cells were transfected with sh-DUXAP8 or empty vector. The cells were then collected, washed with phosphate-buffered saline (PBS), and resuspended at 2 × 10^7^ cells/mL. Each mouse received 100 μL cell suspensions in the lower right flank (n = 6 mice per group). Tumor growth was examined every 3 days, and tumor volume (V) was determined by measuring the length (L) and width (W) of the tumor with a calliper and calculated using the formula V = (L × W^2^) × 0.5. At day 15 post tumor inoculation, the tumors were removed from all mice.

### RNA immunoprecipitation (RIP)

RNA immunoprecipitation was used to investigate whether DUXAP8 could interact or bind to candidate binding proteins (EZH2 and LSD1) in BxPC-3 cells using the EZMagna RIP kit (Millipore, Billerica, MA, USA). BxPC-3 cells were lysed in complete RIP lysis buffer, and the cell lysate was incubated with magnetic beads conjugated with antibodies against EZH2 and LSD1, or control IgG (Millipore, Bedford, MA, USA) for 6 h at 4 °C. Then, the beads were washed and incubated with proteinase K to remove proteins. Finally, purified RNA was subjected to qRT-PCR to examine the expression of DUXAP8 using specific primers.

### Chromatin immunoprecipitation (ChIP)

ChIP assays were performed using the MagnaChIP Kit (Millipore), as previously described [15]. Precipitated chromatin DNA was recovered and analyzed by qRT-PCR. The primer sequences are shown in Additional file 1: Table S1.

### Western blot assays

Cell protein lysates were separated by 10% sodium dodecyl sulfate polyacrylamide gel electrophoresis (SDSPAGE), transferred to 0.22-μm NC membranes (Sigma), and incubated with specific antibodies. ECL chromogenic substrate was used for quantification by densitometry (Quantity One software; Bio-Rad). Antibodies against GAPDH, CDKN1A and KLF2 were used to detect target proteins (Abcam, Hong Kong, China). Protein bands were quantified by densitometry (Quantity One software; Bio-Rad). GAPDH was used as loading control.

### Immunohistochemistry

Tumor specimens from nude mice were stained with hematoxylin and eosin (H&E) and Ki67 for immunohistochemical evaluation. Anti-Ki67 antibody was purchased from Santa Cruz Biotechnology (Santa Cruz, CA, USA). The staining results were independently scored by the author (LYF) and a pathologist (XCX) to minimize subjectivity.

### Statistical analysis

All statistical analyses were performed using SPSS software 22.0 (SPSS, Chicago, IL, USA). Paired Student’s *t*-test was performed to detect the differential expression of DUXAP8 in PC cancer tissues compared with adjacent normal tissues. The relationship between DUXAP8 and clinicopathologic characteristics was evaluated using Chi square test. Survival data were analyzed using Kaplan–Meier analysis, followed by log-rank test. Pairwise comparison, multiple group comparison and correlation analyses were conducted with paired Student’s *t*-test, two-way ANOVA, linear regression test and Pearson test using Graph Pad Prism 5 software (Graph Pad software Inc.), respectively. All data are presented as mean ± SD. Differences were considered significant if *P* < 0.05 (2-sided).

## Results

### DUXAP8 is upregulated in PDAC tissues and predicts shorter OS

**Fig. 1:**
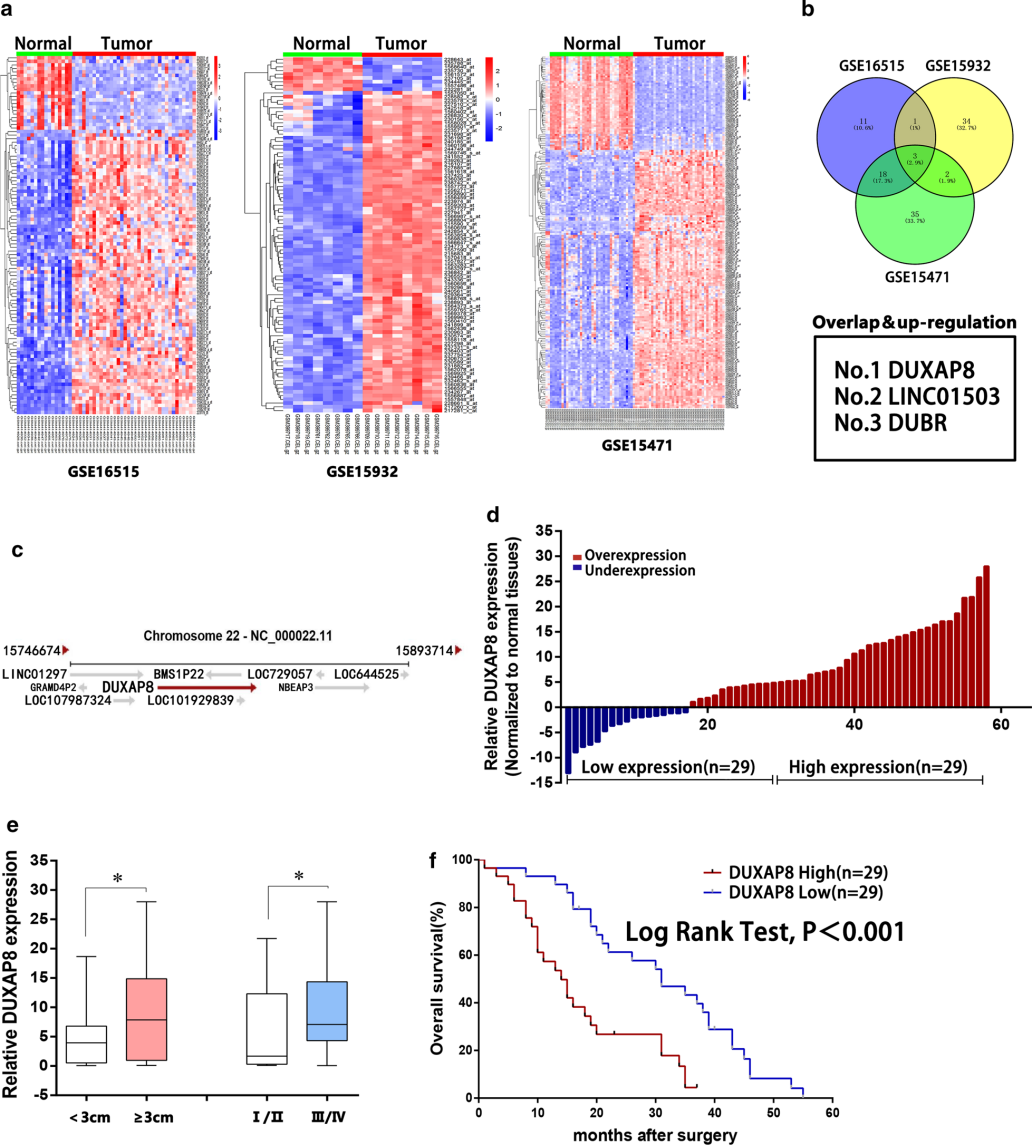
Relative expression of DUXAP8 in pancreatic cancer tissues and its clinical significance. **a** Hierarchical clustering analysis of differentially expressed lncRNAs (fold change > 2; *P* < 0.05) in pancreatic cancer and normal tissues. **b** Overlap of dysregulated lncRNAs in three GEO datasets. **c** The DUXAP8 gene is located at the chromosomal locus 22q11.1 and encodes a 2107-bp transcript (https://www.ncbi.nlm.nih.gov). **d** Relative expression of DUXAP8 in pancreatic cancer tissues compared with paired adjacent normal tissues (n = 58), and DUXAP8 expression was classified into two groups. **e** DUXAP8 expression was significantly higher in patients with a larger tumor size and a more advanced pathological stage (shown as ΔCT). **f** Patients with high DUXAP8 expression had significantly shorter overall survival than patients with low DUXAP8 expression (*P* < 0.001, log rank test). Bars: s.d., *P < 0.05, **P < 0.01

To identify the lncRNAs involved in pancreatic tumorigenesis, we performed an integrative analysis of pancreatic cancer microarray profile comprising GSE16515, GSE15932 and GSE15471 from GEO datasets. A total of 33 lncRNAs were upregulated in GSE16515 datasets, 40 in GSE15932 datasets, and 58 in GSE15471 datasets (fold change > 2.0, *P* < 0.05; Fig. 1a). However, among all the upregulated lncRNAs, only three lncRNAs were consistently upregulated across all the datasets (Fig. 1a, b). Subsequently, considering the expression level of candidate lncRNAs in pancreatic cancer, we focused on the pseudogene derived lncRNA *DUXAP8* for subsequent study. *DUXAP8* is located at the chromosomal locus 22q11.1 and encodes a 2107-bp transcript (Fig. 1c). Next, to validate the analysis results, we examined *DUXAP8* expression in a cohort of 58 paired PDAC and adjacent normal tissues and found upregulated DUXAP8 expression in PDAC tissues *versus* adjacent normal tissues (Fig. 1d).

We further investigated the relationship between *DUXAP8* expression and clinicopathologic variables by using median *DUXAP8* expression to categorize the patients into the high (n = 29) and low (n = 29) expression group. A Chi square test was then performed to evaluate the clinicopathologic variables between the two groups. As shown in Table 1, patients with high and low DUXAP8 expression differed significantly in tumor size (*P *= 0.033) and TNM stages (*P *= 0.0034) (Fig. 1e). The median OS in the low DUXAP8 expression group was 31 months (range 1–55 months) vs. 14 months (range 1–39 months) in the high DUXAP8 expression group (*P* < 0.001; HR = 2.458, 95% CI 1.902–6.439) (Fig. 1f).

### DUXAP8 downregulation inhibits proliferation and induces apoptosis in pancreatic cancer cells

**Fig. 2:**
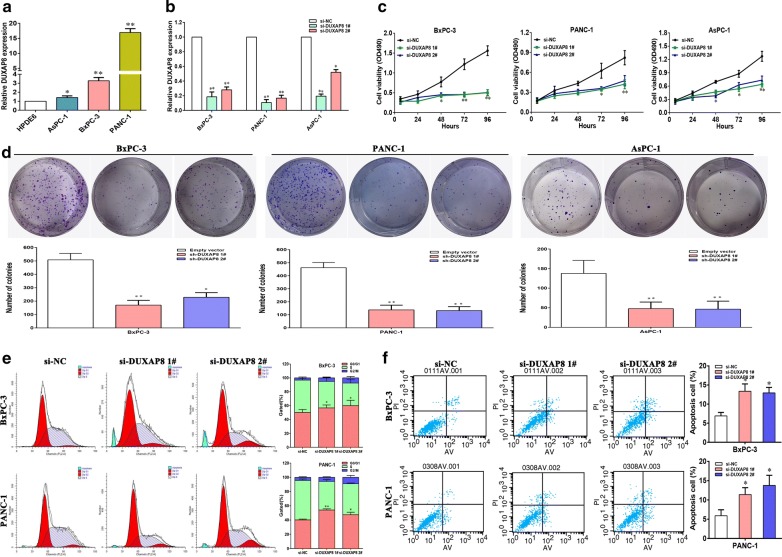
Effects of DUXAP8 on PC proliferation and apoptosis in vitro. **a** Analysis of DUXAP8 expression levels in PC cell lines (AsPC-1, BxPC-3 and PANC-1) compared with a normal pancreatic cell line (HPDE6) by qPCR. Data are shown as fold change (2−ΔΔCT) and the mean ± SD from three independent experiments. **b** DUXAP8 knockdown in PC cells transfected with si-DUXAP8 1# or si-DUXAP8 2#. **c** MTT assays were performed to determine the cell viability of AsPC-1, BxPC-3 and PANC-1 cells after the transfection of siRNA against DUXAP8. **d** Representative results of the colony formation of PC cells transfected with the siRNA of DUXAP8. **e** Flow cytometry assays were performed to analysis the cell cycle progression when BxPC-3 and PANC-1 cells transfected with siRNA against DUXAP8. **f** Flow cytometry assays were performed to analysis the cell apoptotic in siRNA-transfected BxPC-3 and PANC-1 cells. Representative images and data based on three independent experiments. Bars: s.d, *P < 0.05, **P < 0.01

To investigate the biological function of DUXAP8 in pancreatic cancer cells, we first evaluated DUXAP8 expression in pancreatic cancer cells by qRT-PCR assays. Higher DUXAP8 levels were observed in pancreatic cells (AsPC-1, BxPC-3 and PANC-1) *versus* normal HPDE6 cells (Fig. 2a). Furthermore knocking down endogenous DUXAP8 expression in AsPC-1, BxPC-3 and PANC-1 cells with siRNAs reduced DUXAP8 expression by 81.4%, 89.4% and 80.6%, respectively (Fig. 2b). MTT assays confirmed that knockdown of *DUXAP8* suppressed the growth of pancreatic cancer cells (Fig. 2c). Consistently, colony formation assays showed that downregulation of DUXAP8 inhibited cell proliferation ability of AsPC-1, BxPC-3 and PANC-1 (Fig. 2d). In addition, flow cytometry indicated that knockdown of DUXAP8 expression arrested the cell cycle at the G1/G0 phase and induced apoptosis in BxPC-3 and PANC-1 cells (Fig. 2e, f).

### DUXAP8 downregulation inhibits the growth of pancreatic cancer xenografts

**Fig. 3:**
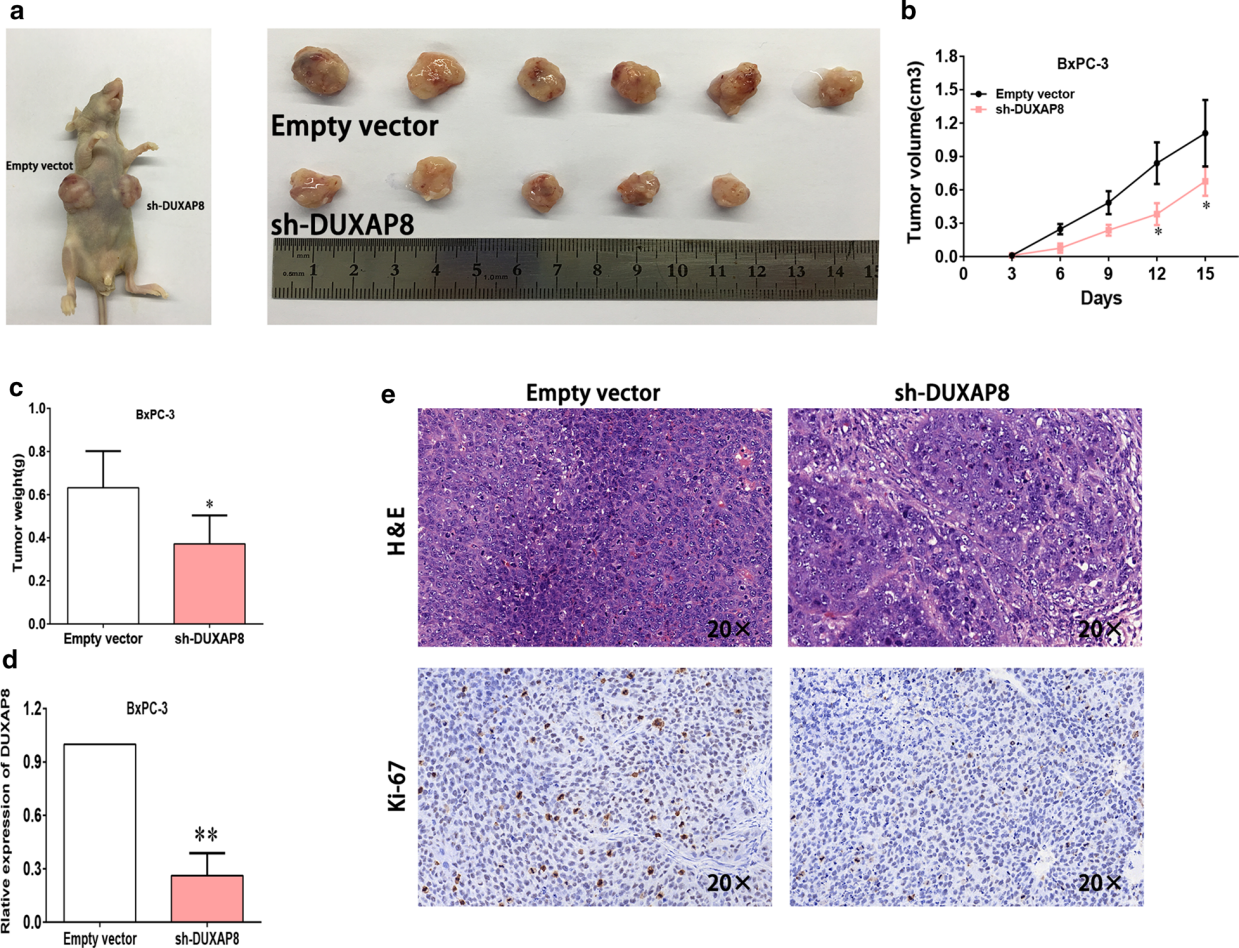
The silencing of DUXAP8 inhibited PC growth in vivo. **a** BxPC-3 cells with stable DUXAP8 knockdown were used for the in vivo study. Nude mice carrying tumors from respective groups are shown. **b** Tumor volumes were calculated after injection every 3 days. **c** Tumor weights from two groups are represented. **d** Images of HE staining and immunohistochemistry of the xenograft tumors. Representative Ki-67 protein levels in xenograft tumors as evaluated by IHC. Representative images and data based on three independent experiments. **e** qPCR was performed to detect the average expression of DUXAP8 in xenograft tumors (n = 6). Bars: s.d, *P < 0.05, **P < 0.01

We further investigated the effect of DUXAP8 on the growth of BxPC-3 xenograft. sh-DUXAP8 markedly suppressed the growth of the xenografts with a significantly lower tumor volume and weight *versus* the controls (Fig. 3a–c). As shown in Fig. 3d, qRT-PCR assays showed lower DUXAP8 levels in the tumor tissues derived from sh-DUXAP8-transfected cells (Fig. 3d). Moreover, immunohistochemistry revealed that tumor xenografts bearing sh-DUXAP8-transfected cells had weaker Ki67 staining than the control xenografts (Fig. 3e). These data demonstrated that DUXAP8 downregulation significantly inhibited the growth of tumor xenograft.

### DUXAP8 serves as modular scaffold for EZH2 and LSD1, thus epigenetically silencing of CDKN1A and KLF2

**Fig. 4:**
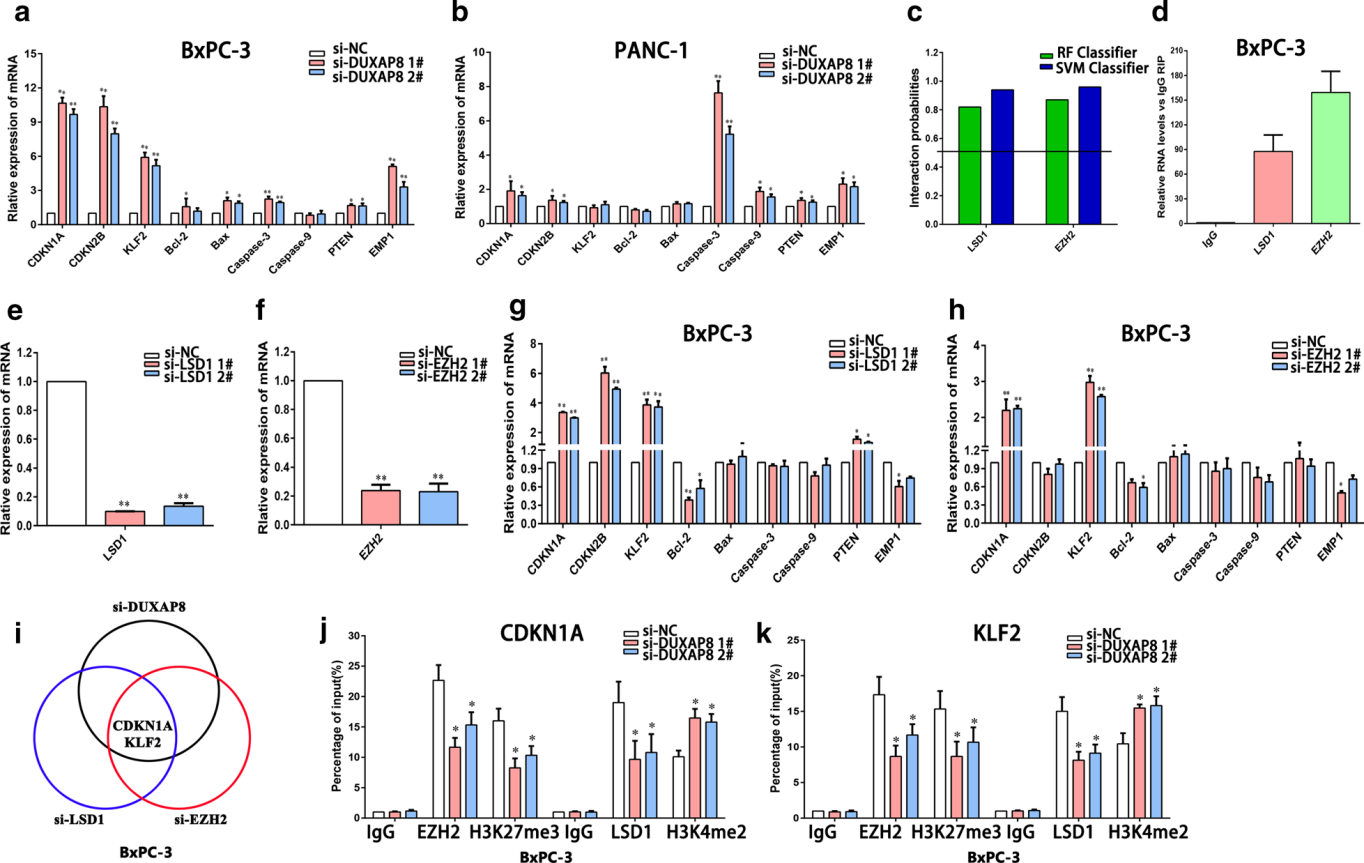
DUXAP8 epigenetically silences CDKN1A and KLF2 transcription by binding with EZH2. **a**, **b** The levels of CDKN1A, CDKN2B, KLF2, Bcl-2, Bax, Caspase-3, Caspase-9, PTEN and EMP1 mRNA were detected by qPCR upon knockdown of DUXAP8 in BxPC-3 and PANC-1 cells. **c** Bioinformatic analysis to predict the interaction probabilities of DUXAP8 and RNA binding proteins via RNA–protein interaction prediction (http://pridb.gdcb.iastate.edu/RPISeq/). Predictions with probabilities > 0.5 were considered positive. RPISeq predictions are based on Random Forest (RF) or Support Vector Machine (SVM). **d.** RIP assays were performed in BxPC-3 cells and the coprecipitated RNA was subjected to qPCR for DUXAP8. **e**, **f** LSD1 and EZH2 expression levels were determined by qPCR when LSD1 or EZH2 were knocked down in BxPC-3 cells. **g**, **h** The levels of CDKN1A, CDKN2B, KLF2, Bcl-2, Bax, Caspase-3, Caspase-9, PTEN and EMP1 mRNA after knockdown of LSD1 or EZH2 in BxPC-3 cells. **i** Overlap of up-regulated genes after knockdown of DUXAP8, LSD1 and EZH2. **j** ChIP-qPCR of H3K4me2 and LSD1, H3K27me3 and EZH2 of the promoter region of the CDKN1A locus after siRNA treatment targeting si-NC or si-DUXAP8 in BxPC-3 cells. **k** ChIP-qPCR of H3K4me2 and LSD1, H3K27me3 and EZH2 of the promoter region of the KLF2 locus after siRNA treatment targeting si-NC or si-DUXAP8 in BxPC-3 cells. Bars: s.d, *P < 0.05, **P < 0.01

Generally, lncRNAs are involved in regulating cancer cell phenotypes via activating oncogenes or inhibiting tumor suppressors by interacting with specific RNA-binding proteins. To investigate the molecular mechanism of DUXAP8 in pancreatic cancer cells, we examined the effects of DUXAP8 knockdown on a total of 9 different cell proliferation related transcripts in BxPC-3 and PANC-1 cells (Fig. 4a, b). We found that DUXAP8 downregulation significantly increased the mRNA transcript levels of *CDKN1A*, *CDKN2B*, *KLF2*, *Bax*, *caspase*-*3*, *caspase*-*9*, *PTEN* and *EMP1* (Fig. 4a, b). Furthermore, bioinformatics analysis indicated two chromatin modifiers, EZH2 (H3K27me3) and LSD1 (H3K4me2) (http://pridb.gdcb.iastate.edu/RPISeq/references.php), as binding proteins for DUXAP8 (Fig. 4c). RIP assays showed that DUXAP8 could directly bind to EZH2 and LSD1 in BxPC-3 cells (Fig. 4d). To further investigate candidate genes involved in DUXAP8 function, we examined the effects of LSD1 or EZH2 knockdown on 9 different cell proliferation related transcripts in BxPC-3 cells (Fig. 4e–h). The result demonstrated that only *CDKN1A* and *KLF2* were consistently upregulated by downregulation of *DUXAP8*, *LSD1* and *EZH2* (Fig. 4i). CDKN1A and KLF2 have been identified as novel tumor suppressors involved in cancer cell proliferation, apoptosis, and invasion [16–18]. Hence, we chose CDKN1A and KLF2 for further experiments.

EZH2 is a negative regulator of transcription via trimethylation of histone 3 lysine 27 (H3K27me3), whereas LSD1 is a negative regulator of transcription via demethylation of histone 3 lysine 4 (H3K4me2) [19, 20]. Therefore, it is likely that DUXAP8 suppresses CDKN1A and KLF2 expression by recruiting EZH2 and LSD1 to the *CDKN1A* and *KLF2* promoter region, leading to trimethylation of H3K27 or demethylation of H3K4 at this region. To verify this hypothesis, we designed three paired primers across the promoter region (2000 bp) of *CDKN1A* and *KLF2*, and conducted ChIP assays by knocking down DUXAP8. We found that knockdown of DUXAP8 decreased binding of EZH2 and H3K27me3 across the CDKN1A and KLF2 promoter region and reduced binding of LSD1 and H3K4me2 (Fig. 4j, k). These data indicated that DUXAP8 could function as a scaffold by binding to EZH2 and LSD1, thus epigenetically silencing CDKN1A and KLF2 in pancreatic cancer cells.

### Silence of CDKN1A and KLF2 is partly involved in the oncogenic function of DUXAP8

**Fig. 5:**
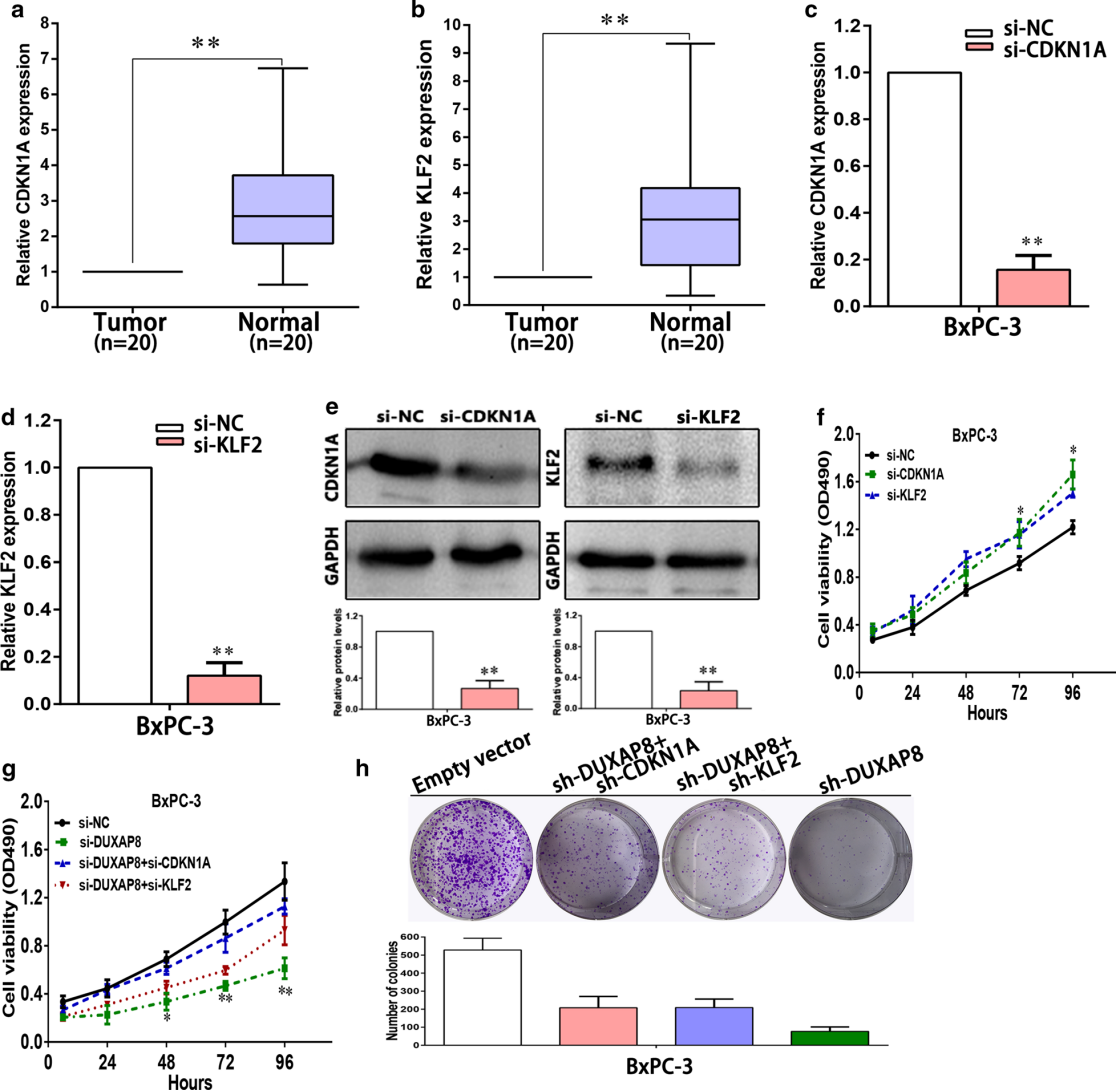
DUXAP8 negatively regulates expression of KLF2 by rescue experiments. **a**, **b** The levels of CDKN1A and KLF2 expression were determined by qPCR in 20 pairs of PC tissues. **c**, **d** The levels of CDKN1A and KLF2 mRNA expression were determined by qPCR when BxPC-3 cells were transfected with si-NC, si-CDKN1A or si-KLF2. **e** The CDKN1A and KLF2 protein levels were determined by Western blot in CDKN1A or KLF2 knockdown BxPC-3 cells. **f** MTT assays were performed to determine the cell viability of BxPC-3 cells after the transfection of siRNA against CDKN1A or KLF2. **g**, **h** MTT and colony formation assays were used to determine the cell proliferation ability for BxPC-3 cells transfected with si-NC and si-DUXAP8 and co-transfected with si-DUXAP8, si-CDKN1A or si-DUXAP8, si-KLF2. Representative images and data based on three independent experiments. Bars: s.d, *P < 0.05, **P < 0.01

qRT-PCR analysis using 20 pairs of pancreatic cancer and adjacent normal tissues showed significantly higher CDKN1A and KLF2 expression in the cancer tissues (Fig. 5a, b). To validate the influence of CDKN1A and KLF2 on cellular proliferation of pancreatic cancer cells, we knocked down *CDKN1A* and *KLF2* expression in BxPC-3 cells (Fig. 5c–e). MTT assays revealed that knockdown of *CDKN1A* or *KLF2* expression could promote cellular proliferation (Fig. 5f). These data showed that CDKN1A and KLF2 could inhibit proliferation of BxPC-3 cells, which was contrary to the effects of DUXAP8 downregulation in pancreatic cancer cells.

Next, to further investigate whether CDKN1A and KLF2 are involved in the enhanced cellular proliferation induced by DUXAP8 in pancreatic cancer cells, we performed rescue experiments. BxPC-3 cells were co-transfected with si-DUXAP8, si-CDKN1A or si-KLF2. MTT and colony formation assays showed that the proliferation ability of BxPC-3 cells co-transfected with si-DUXAP8, si-CDKN1A or si-KLF2 was improved *versus* BxPC-3 cells transfected with si-DUXAP8 (Fig. 5g, h). These results indicated that DUXAP8 exerted oncogenic effect on pancreatic cancer cells partly via repressing CDKN1A and KLF2 expression.

## Discussion

Recently, it has become increasingly apparent that many pseudogenes are transcribed into long non-coding RNAs with potential biological functions [6, 21]. The pseudogene derived lncRNA DUXAP8 was initially reported by Ma et al. [13]. Recent studies indicated that DUXAP8 has a critical role in non-small-cell lung cancer cell proliferation and invasion by epigenetically silencing EGR1 and RHOB [13]. However, the clinical significance and functional role of DUXAP8 expression in pancreatic cancer has not been reported yet. In this study, we identified the pseudogene derived lncRNA DUXAP8 as a novel modulator of pancreatic cancer progression. Our finding indicated that DUXAP8 may serve as a potential therapeutic target for intervention against pancreatic cancer. Importantly, DUXAP8 was significantly upregulated in pancreatic cancer tissues and cells. DUXAP8 upregulation was also associated with larger tumor size, advanced pathologic stage and shorter OS of pancreatic cancer patients. Consistently with these circumstantial findings, knockdown of DUXAP8 remarkably inhibited pancreatic cancer cell proliferation, induced apoptosis, and promoted cell cycle arrest. DUXAP8 induced increase in pancreatic cancer cell proliferation and tumorigenesis may be partly mediated via epigenetically silencing *CDKN1A* and *KLF2* transcription by binding with EZH2 and LSD1. To conclude, the current study indicated that pseudogene derived lncRNA DUXAP8 could act as a non-coding oncogene in PC tumorigenesis.

**Fig. 6:**
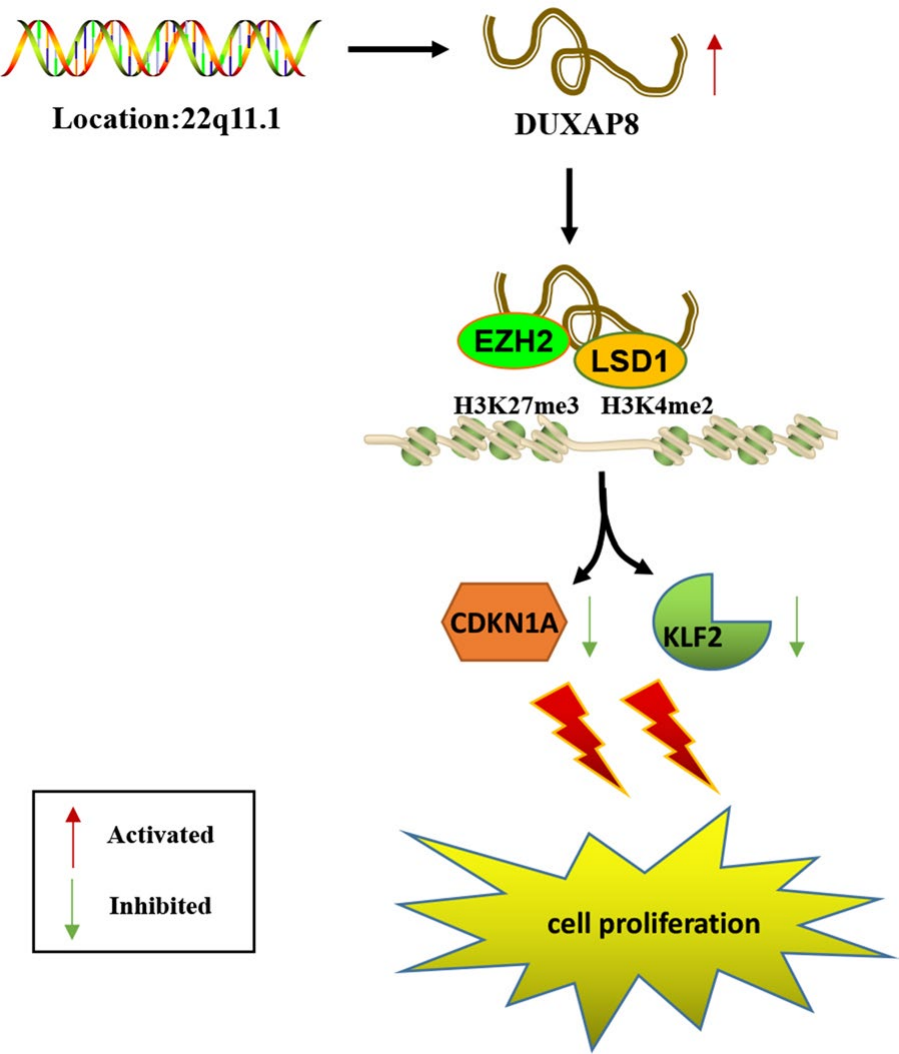
Proposed model for the action of DUXAP8

It has been confirmed that many pseudogenes derived lncRNAs could regulate target gene expression through interacting with RNA binding proteins, such as PRC2, SUV39H1, and LSD1 [12, 13, 22, 23]. EZH2, an important catalytic subunit of PRC2, functions as a histone methyltransferase that specifically induces histone H3 lysine 27 trimethylation (H3K27me3) to the target genes [24]. EZH2 overexpression plays a key role in human oncogenesis and progression [19, 25]. LSD1, also known as KDM1A, is a histone demethylase that specifically demethylates mono- and di-methylated lysine 4 of histone H3 (H3K4me2) [26]. Accumulating evidence indicates that LSD1 could be a novel regulator of cellular process: overexpression of LSD1 has been shown in many cancers including pancreatic cancer [27–29]. In our previous studies, we demonstrated that lncRNA IRAIN suppressed apoptosis and promoted cellular proliferation by binding to LSD1 and EZH2 in pancreatic cancer [30]. Interestingly, in this study, our findings showed that DUXAP8 mediated the oncogenic effects partially through its epigenetic silencing of CDKN1A and KLF2 expression by binding with EZH2 and LSD1 (Fig. 6).

## Conclusions

In summary, we reported a crucial tumor-promoting pseudogene derived lncRNA DUXAP8 in pancreatic cancer, which may serve as a new therapeutic target for pancreatic cancer. Whether DUXAP8 could regulate other downstream target genes and its underlying regulatory mechanisms require further investigation.

## Additional file

**Additional file 1.** Primer sequences and siRNA sequences used in the study.

## Abbreviations

lncRNAs: long non-coding RNAs
DUXAP8: double homeobox A pseudogene 8
KLF2: Kruppel-like factor 2
EZH2: enhancer of zeste homolog 2
LSD1: lysine-specific demethylase 1
PRC2: polycomb repressive complex 2
qRT-PCR: quantitative reverse transcription polymerase chain reaction
TNM: tumor-node-metastasis
siRNA: small interfering RNA
